# A Novel Homozygous CYP19A1 Gene Mutation Causing Aromatase Deficiency

**DOI:** 10.7759/cureus.22059

**Published:** 2022-02-09

**Authors:** Deep Hathi, Soumik Goswami, Nilanjan Sengupta, Arjun Baidya

**Affiliations:** 1 Department of Endocrinology, Nil Ratan Sircar Medical College, Kolkata, IND

**Keywords:** novel mutation, maternal virilization, ambiguous genitalia, aromatase deficiency, cyp19a1 gene

## Abstract

Aromatase deficiency is a rare autosomal recessive disorder and its exact prevalence is not known. Aromatase enzyme catalyzes the conversion of androgens to estrogens in gonadal and extra-gonadal tissues. Deficiency of aromatase enzyme can lead to ambiguous genitalia in a female child and maternal virilization during pregnancy due to raised androgen levels in the mother. A 10-month-old child was referred to our outpatient department for the evaluation of ambiguous genitalia. There was a history of maternal virilization during pregnancy. Karyotype of the child was 46XX. Congenital adrenal hyperplasia was ruled out as serum cortisol, plasma adrenocorticotropic hormone, and 17-hydroxyprogesterone were within normal limits. Hormonal assays showed elevated follicle-stimulating hormone and luteinizing hormone, with raised testosterone and low estradiol levels. Based on these findings, aromatase deficiency was suspected. A novel homozygous mutation c.1376delA located on exon 10 was identified on the *CYP19A1* gene. We identified a novel mutation in the *CYP19A1* gene in a patient who presented with ambiguous genitalia and maternal virilization during pregnancy.

## Introduction

Aromatase enzyme is a part of CytP450 superfamily, which is encoded by the *CYP19A1* gene and is located on chromosome 15q21.1 [1]. Aromatase is involved in conversion of the three androgenic precursors, which includes androstenedione, testosterone, and 16-α-hydroxy dehydroepiandrosterone sulphate to estrone, estradiol, and estriol, respectively [1-3]. The enzyme aromatase is expressed in various body tissues including the placenta, adipose tissue, skin, ovaries, testes, and brain. Deficiency of aromatase enzyme in the fetus causes androgen levels to rise in both the mother and fetus. This results in signs of maternal virilization, including acne, hirsutism, clitoromegaly, and deepening of voice, and can cause significant virilization of external genitalia in a female child [4].

## Case presentation

**Figure 1:**
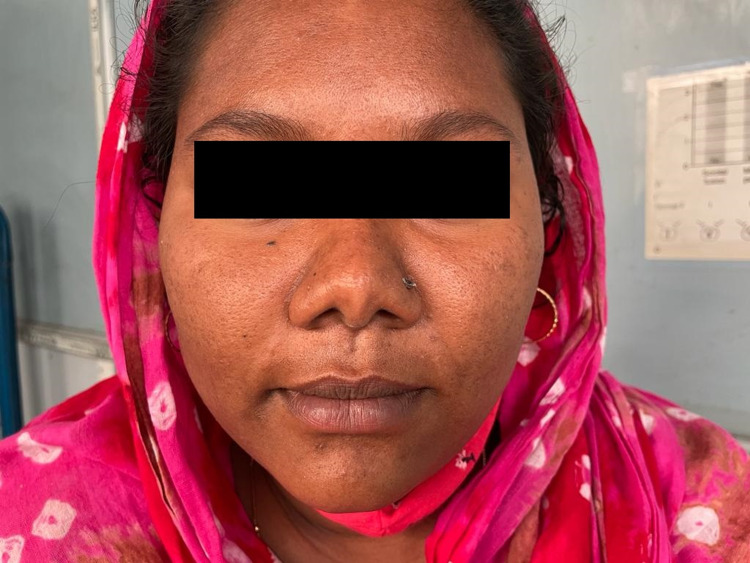
Clinical picture of mother with features of virilsation in form of acne.

**Figure 2:**
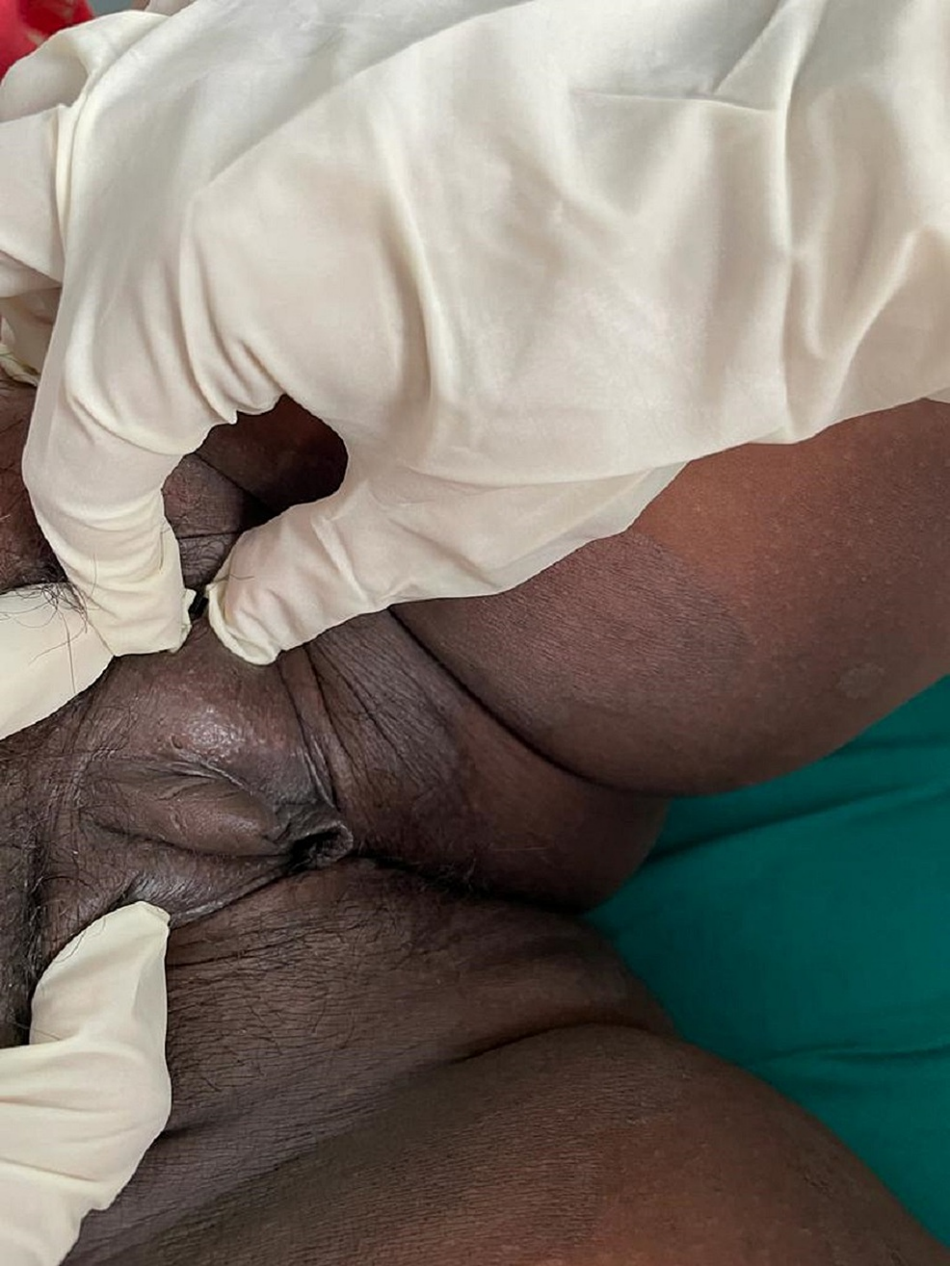
Persistent clitoromegaly in the mother in the post-partum period

We report a case of a 10-month-old child with no sex assigned at birth but reared as a male who presented to our outpatient department with the child’s mother complaining of ambiguous genitalia since birth in the form of clitoromegaly. The child was the first born of a consanguineous marriage and was born via full-term normal vaginal delivery with a birth weight of 2.8 kg. There was no history suggestive of salt-wasting crisis in the form of diarrhea, vomiting, hypotension, hypoglycemia, and lethargy in the early post-natal period. There was no history of hyperpigmentation of genitals, nipples, palms, soles, or oral mucosa. There was a history of maternal virilization during the antenatal period, which was noticed during sixth month of gestation in the form of hirsutism, acne, deepening of voice, and clitoromegaly. There was no history of maternal exposure to androgens. In the post-partum period, the child’s mother noticed partial improvement of virilization in the form of lessening of hirsutism and acne (Figure 1), although deepening of voice and clitoromegaly were persisting (Figure 2).

**Figure 3:**
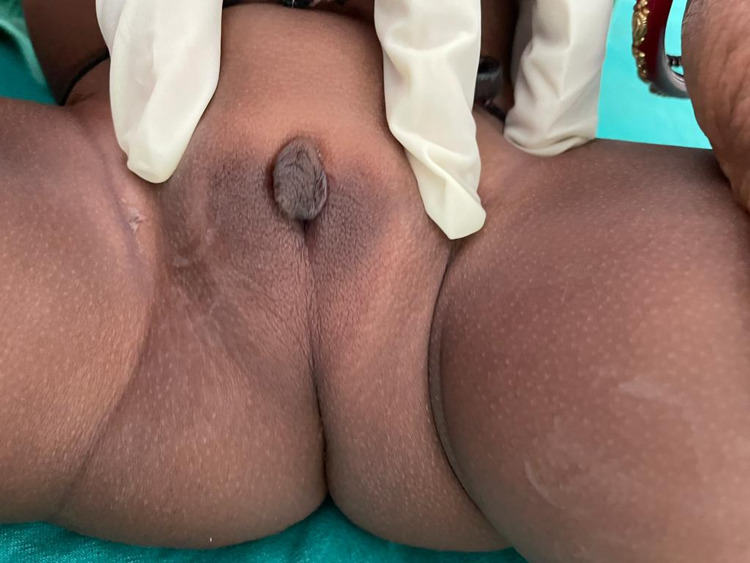
External genitalia of the child (no palpable gonads, fused labioscrotal folds, single urethral opening, and phallus-like structure [Prader stage 4])

**Figure 4:**
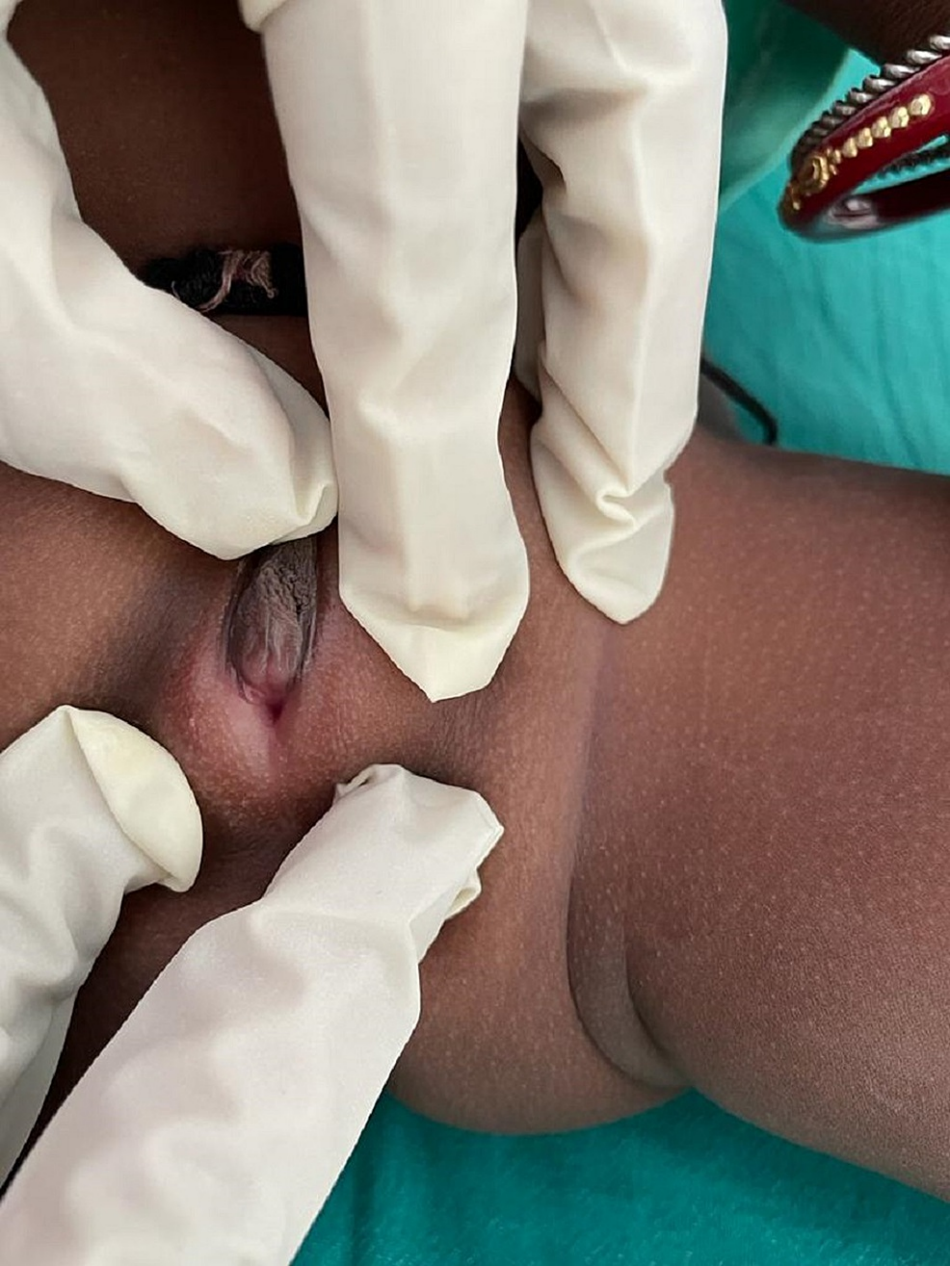
External genitalia of the child

On examination, the child had a pulse rate of 108 per minute and a blood pressure of 98/60 mm Hg. Body weight was 7 kg, and length was 63 cm. There was no evidence of hyperpigmentation of the external genitalia, palms, and oral mucosa. External genitalia examination revealed the absence of palpable gonads, fused labioscrotal folds, along with a phallus of 1.5 cm, single urethral opening at the junction of phallus with labioscrotal folds (Prader stage 4) (Figures 3, 4). Other systemic examination was within normal limit. Hormonal evaluation of the child is summarized in Table 1. Karyotype of the child was 46 XX. Ultrasound of the pelvic region showed uterus-like structure with no evidence of testicular structure. We also investigated the mother simultaneously, and hormonal evaluation was not significant except for a mildly elevated basal 8 a.m. testosterone level of 92 ng/dL (normal <60 ng/dL). Ultrasound of the pelvic region of the mother was within the normal limit.

**Table 1:** Summarizing the hormonal profile of the child DHEAS, dehydroepiandrosterone; FSH, follicle-stimulating hormone;LH, luteinizing hormone; TSH, thyroid-stimulating hormone

Parameter	Value	Reference range
FSH (miu/mL)	113.4	2.5-10.2 mIU/mL
LH (miu/mL)	28.5	1.9-12.5 mIU/mL
TSH (µIU/mL)	3.1	0.4-4.5 µIU/mL
Estradiol (pg/mL)	<10	<10 pg/mL
Testosterone (ng/dL)	50.4	<9 ng/dL
Cortisol (ug/dL)	24.9	8-25 ug/dL
DHEAS (ug/dL)	<15	
17-OH progesterone (ng/mL)	0.249	0.07-1.7 ng/dL

A provisional diagnosis of aromatase enzyme deficiency was made owing to significant history of maternal virilization, biochemical evidence of primary gonadal failure during mini-puberty, and decreased estradiol levels with raised total testosterone levels in the child. One of the closest differential diagnosis in this case was P450 oxidoreductase deficiency; however, there was no obvious bony deformity on clinical examination. Clinical exome sequencing was performed for the child for confirming the diagnosis, which showed a novel mutation of the *CYP19A1* gene. The variant was c.1376delA located on exon 10. This variant has not been reported till date in the literature. Based on the available literature, we have planned to start low-dose estrogen therapy (0.25 mg/day) from the age of two years in this child. Till date, three case reports of *CYP19A1* mutation in Indian origin patients have been reported. The first child was a three-year-old child who presented with ambiguous genitalia, whereas other two were 14-year-old adolescent girls who presented with isolated clitoromegaly.

## Discussion

Aromatase deficiency is a rare condition caused due to a defect in the *CYP19A1* gene leading to impairment in conversion of androgens to estrogens. It was first described by Shozu et al. [5] and has an autosomal recessive inheritance pattern. Very few cases of aromatase deficiency have been reported till date, with more than 30 mutations known to occur in the *CYP19A1* gene [1,2,4,6-9]. Majority of this mutation are located in exons 9 and 10 [9]. Our patient has mutation in exon 10 of the *CYP19A1* gene.

Patients with aromatase deficiency have a wide spectrum of manifestations depending on gender, age, and enzymatic activity [1]. Aromatase deficiency results in increased intrauterine androgen concentration, which can cause varying degrees of virilization in the external genitalia in a girl child. However, external genitalia in boys do not show any change. Our patient presented with ambiguous genitalia (Prader stage 4) and had a karyotype of 46XX. There were no symptoms of aromatase deficiency during infancy and early childhood (particularly in boys), whereas females can present with abdominal symptoms due to ovarian cysts [3]. In adolescent girls, aromatase deficiency causes delayed puberty, hypergonadotropic hypogonadism, multi-cystic ovaries, and primary amenorrhea due to lack of estrogen. Patients can also present with signs of virilization such as acne, deepening of voice, hirsutism, and clitoromegaly due to excess of androgens [1,2,10,11]. Lack of estrogen can also lead to delayed epiphyseal closure, eunuchoid body proportion, osteopenia, and osteoporosis in both the sexes [12].

In most children with aromatase deficiency, usually a history of maternal virilization is present either in the early (12 weeks) or later (upto 30 weeks) part of pregnancy [11,13]. The proposed mechanism for this manifestation is the presence of high levels of non-aromatized feto-placental and maternal androgen precursors, which are converted to testosterone in the placenta and in peripheral maternal tissues, leading to maternal virilization. Post-delivery, the features of virilization in the mother gradually resolve and androgen levels return to normal [1]. In our patient, there was a history of maternal virilization from sixth month of gestation in the form of acne, hirsutism, clitoromegaly, and deepening of voice. Post-delivery, the mother had complete resolution of acne and hirsutism, although deepening of voice and clitoromegaly were persistent.

Hormonal studies have shown that in the first two years of life, both basal and gonadotropin-releasing hormone (GnRH)-stimulated FSH levels tend to be higher in girls with aromatase deficiency as compared with a normal child. However, estradiol and estrone levels are considerably low during the same period [13,14]. Similarly basal luteinizing hormone (LH) tends to be normal or mildly raised during infancy. Our patient had elevated follicle-stimulating hormone (FSH) and LH levels since birth.

Published literature has limited data on effects of early initiation of estrogen replacement to prevent estrogen deficiency in patients with aromatase deficiency. Also, there is lack of consensus on the initial dose of estrogen to be used and the age to start estrogen therapy in such patients. A single study looked at the effect of estrogen therapy on longitudinal growth, bone age maturation, bone density, and multicystic ovaries in a girl with aromatase deficiency who was initiated with estrogen replacement therapy at the age of 3.5 years and followed up till 15 years of age, which concluded that estrogen was required for normal growth, pituitary-gonadotropin feedback development, and bone maturation in puberty as well as early childhood [3]. A review of treatment of aromatase deficiency recommends that the lowest dose of estrogen therapy can be initiated as early as two years of age for the development of ovarian cysts and to avoid early breast development and bone maturation. The recommended dose of estrogen to start with is conjugated estrogen with a dose of 0.15mg/day or alternate day or with micronized estradiol (0.25 mg/day or alternate day), and the dose should be titrated so as to maintain suppressed FSH and LH [4]. We have planned to initiate low-dose estrogen replacement therapy in our patient from the age of two years.

## Conclusions

We reported a case with ambiguous genitalia due to aromatase deficiency caused by a novel mutation in the *CYP19A1 *gene. Aromatase deficiency should be kept as a possible diagnosis in patients with 46 XX karyotype presenting with ambiguous genitalia and having a history of maternal virilization.
